# Immobilization of EreB on Acid-Modified Palygorskite for Highly Efficient Degradation of Erythromycin

**DOI:** 10.3390/ijerph191711064

**Published:** 2022-09-04

**Authors:** Shensheng Ni, Chunyu Li, Yicheng Yu, Dongze Niu, Jie Zhu, Dongmin Yin, Chongqing Wang, Wenfan Zhang, Xingmei Jiang, Jianjun Ren

**Affiliations:** 1 Institute of Urban and Rural Mining, Changzhou University, No. 21 Gehu Road, Wujin District, Changzhou 213164, China; 2 National-Local Joint Engineering Research Center for Biomass Refining and High-Quality Utilization, Changzhou University, No. 21 Gehu Road, Wujin District, Changzhou 213164, China; 3 Jiangsu Key Laboratory of Phylogenomics and Comparative Genomics, School of Life Sciences, Jiangsu Normal University, No. 101 Shanghai Road, Tongshan District, Xuzhou 221116, China; 4 Beijing General Station of Animal Husbandry, No. 21 Chaoqian Road, Changping District, Beijing 100101, China; 5 Bijie Institute of Animal Husbandry and Veterinary Sciences, De Gou Ma Jia Yuan, Qixingguan District, Bijie 551700, China

**Keywords:** EreB, erythromycin degradation, immobilization, palygorskite

## Abstract

Erythromycin is one of the most commonly used macrolide antibiotics. However, its pollution of the ecosystem is a significant risk to human health worldwide. Currently, there are no effective and environmentally friendly methods to resolve this issue. Although erythromycin esterase B (EreB) specifically degrades erythromycin, its non-recyclability and fragility limit the large-scale application of this enzyme. In this work, palygorskite was selected as a carrier for enzyme immobilization. The enzyme was attached to palygorskite via a crosslinking reaction to construct an effective erythromycin-degradation material (i.e., EreB@modified palygorskite), which was characterized using FT-IR, SEM, XRD, and Brunauer–Emmett–Teller techniques. The results suggested the successful modification of the material and the loading of the enzyme. The immobilized enzyme had a higher stability over varying temperatures (25–65 °C) and pH values (6.5–10.0) than the free enzyme, and the maximum rate of reaction (V_max_) and the turnover number (k_cat_) of the enzyme increased to 0.01 mM min^−1^ and 169 min^−1^, respectively, according to the enzyme-kinetics measurements. The EreB@modified palygorskite maintained about 45% of its activity after 10 cycles, and degraded erythromycin in polluted water to 20 mg L^−1^ within 300 min. These results indicate that EreB could serve as an effective immobilizing carrier for erythromycin degradation at the industrial scale.

## 1. Introduction

The discovery of antibiotics is one of the most outstanding achievements in the history of microbiology. An antibiotic is a chemical substance that is produced during the growth of some microorganisms that has an inhibitory or killing effect on other pathogenic microorganisms. Antibiotics can be classified as tetracyclines, β-lactams, aminoglycosides, or macrolides [1]. Erythromycin is an example of a macrolide antibiotic [2]. The increasing global population and demand for animal husbandry has resulted in the extensive use, and even abuse, of erythromycin, which threatens living organisms due to its toxicity and the development of resistance [3,4,5]. Every year, more than two million tons of antibiotic fermentation wastes are produced in China [6]. The inadequate disposal of antibiotic residues, such as those eliminated through excretion, and the lack of treatment of urban, industrial, agricultural, and hospital wastes, has resulted in the contamination of natural waters [4,7]. Therefore, erythromycin was included on the Drinking Water Contaminant Candidate List of the US Environmental Protection Agency, and on the watch list of the European Union of substances to be monitored in surface water [8]. Clearly, developing practical approaches to treat erythromycin pollution is essential.

The erythromycin-degradation processes include incineration, hydrothermal treatment, chemical treatment, composting, and exposure to ionizing radiation or microwaves [9,10]. However, these treatments are often costly and create additional environmental issues. Notably, the biological treatment of erythromycin has the advantages of low treatment costs and an absence of secondary pollution. During the past few decades, studies of natural treatment processes have focused on the screening and isolation of erythromycin-degrading bacteria. For example, degradation rates of *Delftia lacustris* and *Penicillium oxalicum* of 45.18% [11] and 84.88%, respectively, were reported. However, the direct use of microorganisms might lead to the enrichment of resistance genes [12]. Enzyme treatment has also been considered a promising approach.

The known erythromycin-degradation enzymes, which include erythromycin esterase A (EreA), erythromycin esterase B (EreB), and Bcr136, are esterases that can effectively degrade erythromycin under mild conditions [13,14]. Notably, EreB has no metal requirement for its activity [14,15,16,17]. In addition, the ready availability of EreB compared with other enzymes makes it a good candidate for erythromycin degradation. However, practical application has been limited by its non-recyclability and fragility.

Immobilization can solve the non-recyclability issue, and EreB synthesis can be optimized by controlling the pH, solvent, and temperature [18]. Palygorskite, which is also known as attapulgite, is a hydrated aluminum magnesium silicate [19] that serves as a natural, inexpensive, and adsorbent clay mineral. Its unique physicochemical characteristics include a large surface area, swellability, and ion exchangeability [20]. These features enable clay minerals to serve as excellent supports for enzyme immobilization. In addition, the compositional and structural characteristics of palygorskite enable its modification by organic, polymeric, and biological molecules [21,22,23], and the functionalization of carriers by different kinds of acids is always used to increase the adsorption of protein [24]. There have been many reports of the successful fixing of esterase or lipase to modified palygorskite. *Pichia pastoris* recombinant cold-adaptive lipase (rePcLip) from *Penicillium cyclopium* was immobilized on modified palygorskite to develop a robust biocatalytic system for biodiesel production from soybean oil. After reusing immobilized rePcLip for eight cycles, the residual activity and biodiesel yield were 71% and 65%, respectively [25]. Fan et al. successfully used modified palygorskite as a carrier to immobilize plant esterase [26]. Compared with the free enzyme, the immobilized enzyme displayed improved thermal, pH, and storage stabilities. Although the above examples indicate the suitability of palygorskite as an immobilization platform for erythromycin-degrading enzymes, there has been no reported research on the enzyme immobilization on palygorskite for the degradation of antibiotics, and specifically erythromycin.

This research aimed to elaborate a method to immobilize EreB on modified palygorskite. The modified palygorskite was obtained after the treatment of palygorskite by HCl [27], followed by crosslinking by glutaraldehyde. Then, EreB was immobilized on the surface of the modified palygorskite under mild conditions to form the EreB@modified palygorskite composite. The immobilization quality was evaluated under different enzyme loadings, pH values, and temperatures. The materials (palygorskite, modified palygorskite, EreB@modified palygorskite) were analyzed using various analytical techniques, such as field-emission scanning electron microscopy (SEM) (Zeiss, Oberkochen, Germany), Fourier transform infrared (FT-IR) spectroscopy, X-ray diffraction (XRD), and Brunauer–Emmett–Teller (BET) surface-area analysis. Moreover, colorimetry and high-performance liquid chromatography (HPLC) were used to assay the enzyme activity to study the composite stability, recyclability, and performance in erythromycin-polluted wastewater.

## 2. Materials and Methods

### 2.1. Materials

All chemicals and reagents were AR grade and were purchased from Sigma-Aldrich (St. Louis, MO, USA). Luria–Bertani (LB) medium containing 10.0 g·L^−1^ peptone, 5.0 g·L^−1^ yeast extract, and 10.0 g·L^−1^ NaCl was used to culture the strain. *Escherichia coli* (*E. coli*) BL21 (DE3) was used as the expression host. Palygorskite clay was obtained from the School of Materials Science and Engineering, Changzhou University.

### 2.2. Gene Cloning, Vector Construction, and Heterogeneous Expression

The gene sequence encoding the EreB enzyme was identified in the NCBI Gene Bank (accession number NG_047768). The plasmid used in this study was synthesized by Sangon Biotech Inc. (Shanghai, China). Recombined *E. coli* BL21 (DE3) carrying the plasmid pET28a was constructed in our laboratory. Agarose gel electrophoresis was used to confirm the plasmid transformation into the recombined *E. coli*. The recombined *E. coli* was inoculated at 37 °C in liquid LB medium containing kanamycin (20 μg·mL^−1^) until the optical density measured at 600 nm reached 0.8–1.0 when the target protein (EreB) was induced using isopropyl-β-D-1-thiogalactopyranoside (IPTG) (final concentration: 1 mmol·L^−1^), followed by incubation at 18 °C overnight [28]. Then, the supernatant and sediment of the recombined *E. coli* were analyzed by sodium dodecyl sulfate–polyacrylamide gel electrophoresis (SDS-PAGE).

All purifications were performed at 4 °C unless otherwise stated. The expressed cells were obtained by centrifugation at 10,000× *g* for 10 min. The cells were concentrated with 50 mL of PBS buffer (pH 7.0, 20 mM) four times, and were then ruptured using ultrasound. The intracellular proteins, including the EreB of the recombined *E. coli*, were released, and the crude enzyme solution was passed through a 0.22 μm filter. The filtrate was eluted through a Ni-NTA Purose column (Qianchun Biotechnology, Shanghai, China) using phosphate buffer solution (PBS) (20 mM, pH 7.4) and an imidazole concentration gradient of 20, 50, 100, 150, 200, 250, 300, 400, and 500 mM. SDS-PAGE was used to verify the purification, and the purified enzyme was concentrated by ultrafiltration.

### 2.3. Palygorskite Modification by Acid and Glutaraldehyde

Palygorskite (15 g) was added to 150 mL of 2 mol L^−1^ hydrochloric acid. The mixture was stirred at 60 °C for 2 h, filtered by a 100-mesh sieve, washed to neutrality by distilled water, dried in a dry oven at 65 °C, pulverized, and passed through a 100-mesh sieve to obtain acid-modified palygorskite. Then, 10 g of acid-modified palygorskite was added to 50 mL of a 15% aqueous solution of 3-methacryloxypropyltrimethoxysilane (KH570), and the pH value was adjusted to 3–4 with hydrochloric acid. The mixture was stirred at room temperature for 12 h, centrifuged, washed, and dried to obtain KH570-coupled palygorskite. Specific amounts of KH570-coupled palygorskite were added to glutaraldehyde solutions of different concentrations. Drying the solutions provided the modified palygorskite [26,29].

### 2.4. Synthesis of EreB@modified Palygorskite and Reaction Optimization

An appropriate amount of modified palygorskite was added to the purified enzyme in PBS, shaken on a water-bath shaker for 5 h, suction-filtered, repeatedly washed with distilled water, freeze-dried for 24 h, and finally stored at low temperature. The optimal conditions for immobilization were identified by systematically varying the amount of glutaraldehyde, the amount of added enzyme, and the temperature.

The recovery rate was determined as follows:

(1) $$\mathrm{Recovery}\;\mathrm{rate}=\frac{\mathrm{amount}\;\mathrm{of}\;\mathrm{enzyme}\;\mathrm{introduced}-\mathrm{amount}\;\mathrm{of}\;\mathrm{enzyme}\;\mathrm{in}\;\mathrm{eluent}}{\mathrm{amount}\;\mathrm{of}\;\mathrm{enzyme}\;\mathrm{introduced}}\times 100\%$$

The protein content in the purified enzyme solution and eluent was determined using the Bradford method [30].

Enzyme activity was determined using *p*-nitrophenyl butyrate (pNP-C4) as the substrate [31]. The reaction was initiated by adding 10 mg of EreB@modified palygorskite to 1 mL of 1 mM pNP-C4 in 10 mM potassium phosphate buffer (pH 7.4) containing 0.1% Triton X-100. The release of p-nitrophenol was determined according to the change in absorbance, which was measured at 405 nm using a UV2600 ultraviolet (UV)–visible spectrophotometer (Shimadzu, Kyoto Japan). One unit of activity was defined as the amount of enzyme releasing 1 μmol of pNP-C4 each minute under the described conditions. Relative activity refers to the unit mass of the successfully immobilized enzyme. In our previous pre-experiment, the optimal conditions of each single factor were obtained (Table S1).

### 2.5. Characterization of EreB@modified Palygorskite

Field-emission SEM, conducted at an accelerating voltage of 5 kV, was used to investigate the particle size and morphology. FT-IR spectra were obtained using a Nicolet iS50 instrument (Thermo Fisher Scientific, Waltham, MA, USA). X-ray-powder-diffraction patterns were obtained using a SmartLab 9 instrument (Rigaku, Tokyo, Japan) with Cu Kα radiation. The specific surface area and pore size distribution were analyzed according to the BET method. The nitrogen adsorption isotherm was obtained at 473 K using a TriStar 3020 instrument (Micromeritics, Norcross, GA, USA).

### 2.6. Determination of Enzyme Activity

*P*-nitrophenyl butyrate was used as the substrate to study the effects of the temperature and pH on the enzyme activity. The enzyme concentration was kept constant for the composite.

The kinetic parameters were obtained by measuring the initial rate of reaction as a function of the erythromycin concentration, and by applying the nonlinear regression of the Michaelis–Menten equation [32], as follows:

(2) $${\;V}_{0}=\frac{V_{\max}\left[S\right]}{K_{M}+\left[S\right]}$$

where V_0_ is the initial catalytic rate, K_M_ is the Michaelis–Menten constant, V_max_ is the maximum rate of reaction, and [S] is the initial substrate concentration.

The reaction solution contained 0.091 nM EreB, and the erythromycin concentration was determined by HPLC (Ultimate 3000; Thermo Fisher Scientific) using a C18 reversed-phase column (ZORBAX SB-C18; 150 × 4.6 mm; 5 μm; Agilent, Santa Clara, CA, USA) with a column temperature of 35 °C. The injection volume was 40 μL. The mobile phase was acetonitrile and K_2_HPO_4_ (0.01 M) at a ratio of 55:45 (*v/v*) and a flow rate of 1 mL·min^−1^. A variable-wavelength UV detector was set at 215 nm [33].

### 2.7. Application of EreB@modified Palygorskite in the Erythromycin Reactor

EreB@modified palygorskite (10 mg) was used for successive batches. The enzymatic activity of the composite was measured as follows. A solution of PBS (pH 7.0) with an erythromycin concentration of 250 mg·L^−1^ was added to 0.01 g of the modified palygorskite composite, and was allowed to react at 35 °C on a water-bath shaker for 10 min. The solution was then centrifuged at 10,000 rpm for 2 min, and the supernatant was collected. An equal volume of methanol was added to terminate the reaction. The erythromycin concentration was then determined by HPLC, as described above. The collected precipitate and equivalent fresh substrate solution were mixed again under the same conditions (10 repeated cycles) to establish reusability.

Application in erythromycin-polluted wastewater was simulated using erythromycin-contaminated water samples taken from a river near a pharmaceutical factory. The erythromycin concentration was adjusted in the laboratory to about 50–100 mg·L^−1^ from the initial concentration (187 ng/L) using pure erythromycin. Then, 20 mg of the composite material was added to 5 mL of the adjusted contaminated water sample, and the mixture was allowed to react at 30 °C on a water-bath shaker. Samples were taken every 30 min, and the erythromycin content was determined by HPLC.

## 3. Results and Discussion

### 3.1. Gene Cloning, Vector Construction, and Heterogeneous Expression of EreB

**Figure 1:**
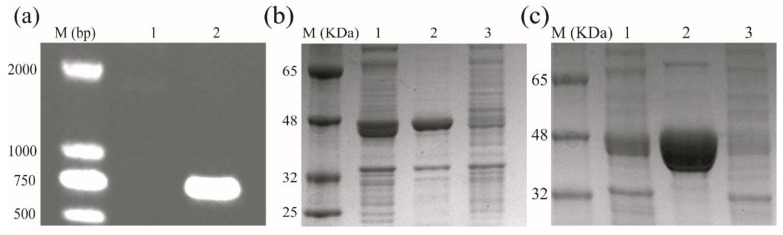
Preparation of purified EreB: (**a**) agarose gel electrophoresis: Lane 1: empty plasmid of *E. coli*; Lane 2: recombinant *E. coli*; (**b**) SDS-PAGE electrophoresis: Lane 1: supernatant of the EreB-expressing cells; Lane 2: sediment of the EreB-expressing cells; Lane 3: empty plasmid of *E. coli*; (**c**) Lane 1: supernatant of the EreB-expressing cells; Lane 2: purified enzyme; Lane 3: empty plasmid of *E. coli*.

PCR primers were designed using the EreB gene. The primers EreB F04 and EreB R847 produced an amplicon of about 740 bp from the recombined *E. coli* (Figure 1a). The target gene was successfully expressed in the recombined *E. coli*; the recombinant protein extracted by SDS-PAGE is shown in Figure 1b. The recombinant protein was the major protein in the supernatant and precipitate in Lanes 1 and 2, respectively, with a molecular weight of approximately 48 kDa. IPTG-induced recombinant protein appeared at 48 kDa, which is consistent with the predicted molecular weight of 48.18 kDa (Geneious 3.0). No recombinant protein was detected in Lane 3, which was the IPTG-induced *E. coli* BL21 (DE3) control. The target protein was detected in the 100, 150, 200, and 250 mM imidazole lanes; the permeate was collected and ultrafiltered to concentrate the enzyme. The purification result is shown in Figure 1c; the purity of the target enzyme is evident in Lane 2.

### 3.2. Optimization of Temperature, Enzyme, and Glutaraldehyde Loadings

**Figure 2:**
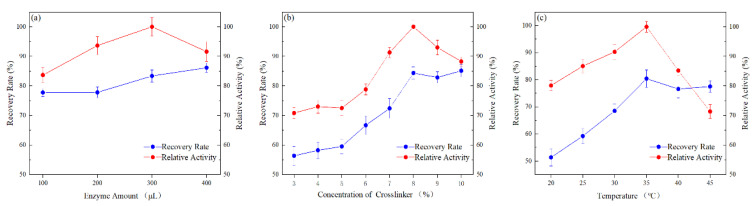
Optimization of: (**a**) enzyme loading, (**b**) crosslinker concentration, and (**c**) temperature.

Single-factor experiments were conducted by varying the amount of glutaraldehyde, the amount of enzyme introduced, and the temperature during immobilization, while keeping all other conditions constant. The recovery and enzyme activity were measured (Figure 2). Modified palygorskite (0.01 g) was added to PBS (20 mM; 2 mL), and was then introduced to 100, 200, 300, and 400 μL of purified enzyme solution (immobilized under room-temperature conditions and pH 7) to study the effect of the enzyme amount (Figure 2a). The recovery rate increased and the activity of the immobilized enzyme first increased and then decreased with the increasing enzyme amount. This behavior reflected the increasing combination of the enzyme with the palygorskite as the amount of added enzyme increased. The activity of the immobilized enzyme reached its maximum when the amount of enzyme was 300 μL. At this point, the active groups on the surface of the palygorskite were nearly saturated, the steric-hindrance effect was enhanced, and it was difficult for additional enzyme molecules to adsorb onto the palygorskite surface. When more enzymes were introduced, the catalytic activity was decreased, and this may be due to the fact that the overloading of EreB causes a multilayered stacking of the enzyme, which blocks the substrate molecules from accessing the internal EreB. Adding more enzymes would have been wasteful. The maximum enzyme loading rate was 39.9 mg·g^−1^.

Glutaraldehyde was used as the crosslinker because it contains two aldehyde groups that can easily react with the amino function of proteins and form crosslinks [34]. Figure 2b shows the effect of the crosslinker amount. Initially, the enzymatic activity of the immobilized enzyme changed slowly, but when the concentration exceeded 5%, the enzymatic activity increased markedly. When the concentration reached 8%, the enzymatic activity of the immobilized enzyme reached its maximum, and the further addition of the crosslinking agent caused the enzyme activity to decline. This was likely due to the covalent bonding of the superfluous crosslinking agent to the active center of the enzyme, which caused the denaturation of the enzyme and thereby reduced the enzyme activity. A contributing factor might be the increased steric hindrance caused by the immobilization of enzyme molecules, which restricted the access of the substrate to the active center.

It is well known that enzymes are very sensitive to temperature. The temperature had a notable influence on the enzyme immobilization. Protein adsorption is an endothermic process. An improper temperature could have a negative impact on the enzyme immobilization. The enzyme was not readily adsorbed onto the palygorskite surface at low temperature. However, too high a temperature destroys the protein molecular structure, with the resulting loss of activity [35]. We chose 35 °C as the immobilization temperature (Figure 2c).

According to the results above, the loading condition of the enzyme was mild, which maintained the high biological activity of the enzyme and showed it to be environmentally friendly. Thus, the result is conducive to industrial-scale application in the future.

### 3.3. Characterization of EreB@modified Palygorskite

**Figure 3:**
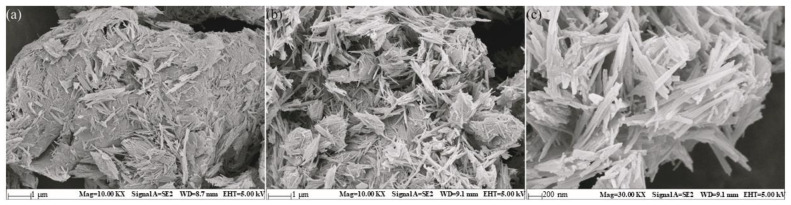
Scanning electron micrographs of: (**a**) palygorskite (10,000×), (**b**) modified palygorskite (10,000×), and (**c**) EreB@modified palygorskite (30,000×).

Figure 3a,b shows that the agglomeration of palygorskite particles increased significantly during the crosslinking reaction with glutaraldehyde. Such crosslinking led to a reduced BET specific surface area, but the fibrous shape of the original palygorskite particles was maintained. After the enzyme was successfully loaded, the surface roughness increased (Figure 3c), possibly due to the loading of enzyme molecules onto the surface; this is consistent with the BET data.

**Figure 4:**
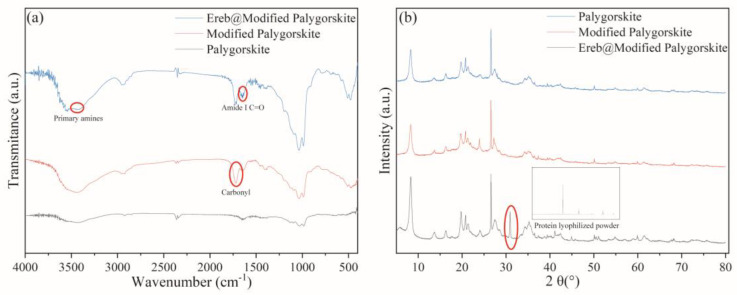
(**a**) Fourier-transform-infrared spectra of palygorskite (black), modified palygorskite (red), and EreB@modified palygorskite (blue). (**b**) X-ray-diffraction patterns of palygorskite (black), modified palygorskite (red), EreB@modified palygorskite (blue), and crude protein lyophilized powder.

The FT-IR spectra (Figure 4a) show vibrations characteristic of proteins at 1640–1660 cm^−1^. These primarily correspond to the amide I C=O stretching modes [36]. In the 3500–3200 cm^−1^ range, two sharp middle-intensity bands were observed due to primary amines, which indicated the presence of proteins in the composite [37]. The peak at 1725 cm^−1^ was attributed to the carbonyl group of glutaraldehyde [38]. These observations confirmed that glutaraldehyde crosslinked to the palygorskite before immobilization.

**Figure 5:**
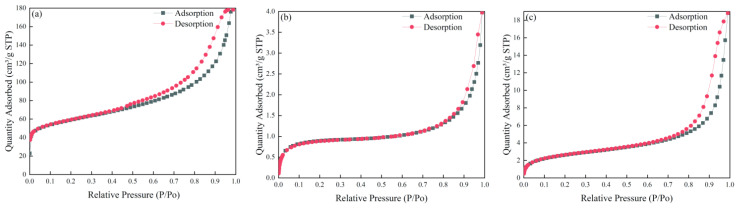
Brunauer–Emmett–Teller analysis of: (**a**) palygorskite, (**b**) modified palygorskite, and (**c**) EreB@modified palygorskite.

The XRD pattern of EreB@modified palygorskite was very similar to those of the original and modified palygorskite (Figure 5). The results confirmed that the crystal structure of the palygorskite was unchanged during the immobilization process. The peak observed at about 2*θ* = 31° was attributed to the enzyme. It also presents the XRD pattern of the crude protein lyophilized powder, which also shows this peak. The XRD data indicate that attaching the enzyme to palygorskite did not alter the crystal structure of the mineral.

Figure 5 presents the BET data for palygorskite, modified palygorskite, and enzyme-loaded modified palygorskite. The sharp upward trend at low pressures observed for all three materials is consistent with the presence of single-layered palygorskite and a microporous surface. Higher pressures were associated with multilayered adsorption, indicating mesoporous properties. Although the complete adsorption and desorption isotherms of the three materials were not coincident, they all displayed H3-type hysteresis loops that suggested slit pores formed by the accumulation of particle flakes and variable pore sizes. As the relative pressure increased, the palygorskite and its two modifications displayed hysteresis loops, but the hysteresis curves of the modified and enzyme-loaded palygorskite materials were notably different. This suggests that the modification and loading of enzymes impacts the palygorskite pore shape and structure.

The data of BET indicate that the modification of palygorskite caused the BET surface area to decrease from 204.9005 to 3.0781 m^2^·g^−1^. Concurrently, the pore volume of the modified palygorskite decreased, indicating that the modified palygorskite had different pore characteristics from the original palygorskite (e.g., blocked pores). The adsorption of the enzyme by the modified palygorskite caused the surface area to increase from 3.0781 to 9.6512 m^2^·g^−1^, and the pore volume to increase from 0.006 to 0.029 cm^3^·g^−1^. These results further supported the successful immobilization of the enzyme onto the surface of the modified palygorskite [39].

### 3.4. Effect of pH and Temperature on Activity

**Figure 6:**
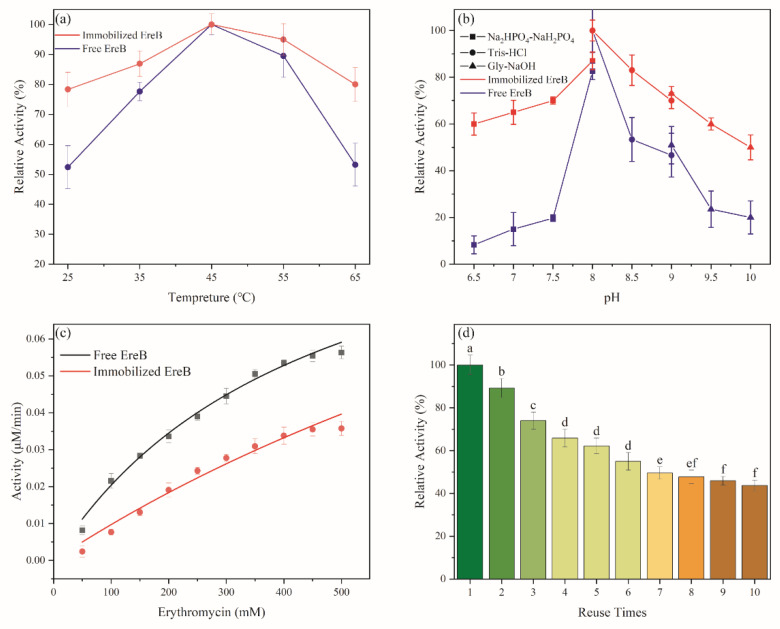
Characterization of the enzymatic properties of free EreB and EreB@modified palygorskite. Effects of: (**a**) temperature and (**b**) pH on the relative activity at their own optimal pH (8.0) and temperature (45 °C), respectively. (**c**) Kinetic analysis of free EreB and immobilized EreB. (**d**) Recycling performance of EreB@modified palygorskite. The letters above different columns addressed not by the same letter are significantly different according to student’s *t* test, (*p* < 0.01).

Thermal stability is one of the important factors affecting the industrial application of enzymes. Figure 6a shows that immobilization did not affect the optimal catalytic temperature, which remained at 45 °C, which is consistent with previous findings [30]. The relative activity was significantly reduced at higher and lower temperatures, which demonstrated that immobilization improved the sensitivity of EreB to temperature. This may be due to the combination of enzyme and carrier molecules, which made the spatial conformation of the enzyme more rigid and thereby caused the immobilized enzyme molecule to resist stretching and opening when heated [18]. In other words, the palygorskite protected the enzyme molecule against protein thermal denaturation.

As is known, the pH could alter the ionization state of protein, and so it is vital to keep the active conformation of enzymes in a broadened pH range. In this study, three buffers were used to determine the effect of the pH (i.e., Na_2_HPO_4_-NaH_2_PO_4_ (pH 6.5–8.0), Tris-HCl (pH 8.0–9.0), and Gly-NaOH (pH 9.0–10.0)). The results presented in Figure 6b show that the optimal activity occurred at pH 8.0 in Tris-HCl. This may be due to the change in the state of the charge of the amino acid in the enzyme’s active center that occurred during immobilization. The immobilized enzyme had a buffering effect, which affected the enzyme’s activity [40]. After immobilization, the environmental tolerance of the enzyme was significantly improved and reuse was capable on account of palygorskite’s ionization and structure protective effect, as mentioned above, which indicated that the immobilized enzyme was more suitable for application at the industrial scale.

### 3.5. Enzyme-Kinetic Parameters of EreB@modified Palygorskite

Figure 6c shows that the activity was reduced after immobilization. The decrease in activity may be due to steric hindrance and conformational effects resulting from the binding of the enzyme to the support [41]. The kinetic parameters were obtained according to the Michaelis–Menten kinetic model (Table 1), and they were used to determine the enzymatic activity of the free and immobilized EreB. The immobilized form exhibited a higher K_M_ (1129.04 mM) than that of the free EreB (438.49 mM), and this phenomenon agreed with the previous report, in which a decreased affinity was aroused after the enzyme immobilization. This phenomenon was owing to the conformation change of the enzyme and mass-transport resistance [42]. The V_max_ of the immobilized enzyme was 0.12 mM·min^−1^, which is nearly the same as that of the free enzyme; this indicated that the maximum reaction rate did not change. It has been suggested that no denaturation of the enzyme occurs during the loading process [43,44]. Although the affinity of EreB decreases after immobilization, the maximum reaction rate increased a little. This means that only the increasing dosage of the composite can make up for the defects caused by immobilization in application, while the degradation rate is simultaneously improved.

### 3.6. Reusability of EreB@modified Palygorskite and Verification of its Degradation Effect in Polluted Wastewater 

**Figure 7:**
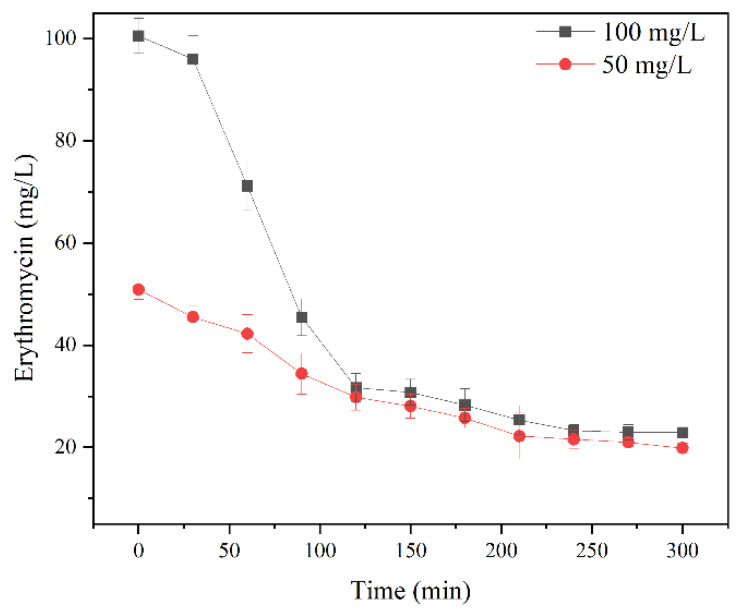
Degradation performance of EreB@modified palygorskite in erythromycin-polluted wastewater containing 50 and 100 mg·L^−1^ erythromycin.

Figure 6d presents the results of the reusability testing. The EreB@modified palygorskite retained about 45% of its activity after 10 cycles. Enzyme deactivation during the recycling process and leakage from constant washing may have contributed to the activity loss [45]. Notably, the activity plummeted in the first few cycles, and then remained relatively stable. This could be because the outermost enzymes were washed away first due to weak binding, and then the remaining enzyme molecules, which were bound more strongly to the surface, resisted washing off [46]. The dynamic process of erythromycin degradation in erythromycin-polluted wastewater by EreB@modified palygorskite was also evaluated (Figure 7). The erythromycin could eventually be degraded to a concentration of about 20 mg·L^−1^, regardless of the initial substrate concentration; the half-life of erythromycin was about 100 min. Such degradability is better than that reported for other erythromycin treatment methods [9,11,31,33]. Therefore, the enhanced tolerance and reusability increased the potential for utilizing the enzyme in harsh conditions.

## 4. Conclusions

In this study, palygorskite, modified by a simple acid treatment followed by crosslinking with glutaraldehyde, was demonstrated to be a suitable EreB carrier for erythromycin degradation. A novel composite was successfully created, which makes it possible to apply the enzyme in environmental engineering. The tolerance to changing environmental conditions and the maximum translation rate were improved. Most importantly, good repeatability was achieved, which is critical for industrial application. The direct binding of erythromycin-degrading enzymes to palygorskite has not been previously reported, and our research findings provide guidance for the industrial processing of erythromycin. We believe that the development of more enzyme-friendly materials will lead to enzyme composite materials becoming essential for antibiotic wastewater treatment.

## Figures and Tables

**Table 1:** Comparison of kinetic parameters of free and immobilized EreB.

Catalyst	K_M_ (mM)	V_max_ (mM min^−1^)	k_cat_ (min^−1^)	k_cat_/K_M_ (min^−1^ mM^−1^)
Free EreB	438.49	0.11	1202.75	2.74
Immobilized EreB	1129.04	0.12	1371.21	1.21

## Data Availability

Not applicable.

## Supplementary Materials

The following supporting information can be downloaded at: https://www.mdpi.com/article/10.3390/ijerph191711064/s1. Table S1: Single factor pre-experiment on optimization of enzyme amount, concentration of crosslinker and temperature; Table S2: Comparison of Brunauer–Emmett–Teller data of palygorskite, modified palygorskite, and EreB@modified palygorskite; Figure S1: Gradient concentration (20, 50, 100, 150, 200, 250, 300, 400, 500 mM) illustrating imidazole purification.
