# Supplementary material for: Immobilization of EreB on Acid-Modified Palygorskite for Highly Efficient Degradation of Erythromycin
Source: Int J Environ Res Public Health. 2022 Sep 4;19(17):11064. doi: 10.3390/ijerph191711064 (PMC9518184; doi:10.3390/ijerph191711064)
Supplement: Supplementary file 1 [file ijerph-19-11064-s001.zip › ijerph-1873842-supplementary.pdf]

Supplementary Materials

Immobilization of EreB on acid-modified palygorskite for highly efficient degradation of erythromycin

Shensheng Ni[1,2], Chunyu Li[1,2], Yicheng Yu[3], Dongze Niu[1,2], Jie Zhu[2], Dongmin Yin[1,2], Chongqing Wang[4], Wenfan Zhang[1,2] , Xingmei Jiang[5] and Jianjun Ren[1,2*]

[1] Institute of Urban and Rural Mining, Changzhou University, No.21 Gehu Road, Wujin District, Changzhou 213164, China.

[2] National-Local Joint Engineering Research Center for Biomass Refining and High-Quality Utilization, Changzhou University, No.21 Gehu Road, Wujin District, Changzhou 213164, China.

[3] Jiangsu Key Laboratory of Phylogenomics and Comparative Genomics, School of Life Sciences, Jiangsu Normal University, No. 101 Shanghai Road, Tongshan District, Xuzhou 221116, China.

[4] Beijing General Station of Animal Husbandry, No. 21 Chaoqian Road, Changping District, Beijing，100101, China.

[5]Bijie Institute of animal husbandry and Veterinary Sciences，De Gou Ma Jia Yuan, qixingguan District, Bijie City, Guizhou Province, 551700, China.

*Correspondence: Jianjun Ren, No.21 Gehu Road, Wujin District, Changzhou, Jiangsu, 213164, China. E-mail: rjj@cczu.edu.cn

Table S1. Single factor pre-experiment on optimization of enzyme amount, concentration of crosslinker and temperature

**Enzyme Amount (μL)**	**Relative Activity (%)**	**Concentration of Crosslinker (%)**	**Relative Activity (%)**	**Temperature (℃)**	**Relative Activity (%)**
Concentration of Crosslinker: 5%; Temperature: 25 ℃		Enzyme Amount: 100 μL; Temperature: 25℃		Enzyme Amount: 100 μL; Concentration of Crosslinker: 5%	
200	90.75±2.08	5	65.23±7.81	30	84.42±6.41
300	100±3.62	6	72.50±2.38	35	100±4.38
400	87.84±4.68	7	86.32±4.23	40	77.21±4.29
500	82.37±7.74	8	100±3.96	45	72.74±5.19
600	75.53±6.29	9	89.06±3.45	50	63.66±5.37

Table S2. Comparison of Brunauer–Emmett–Teller data of palygorskite, modified palygorskite, and EreB@modified palygorskite.

Sample	BET surface area ($m^2\ g^{-1}$)	Pore volume ($cm^3\ g^{-1}$)	Pore size (nm)
Palygorskite	204.9005	0.275876	5.3856
Modified palygorskite	3.0781	0.006151	7.9938
EreB@modified palygorskite	9.6512	0.029116	19.6135

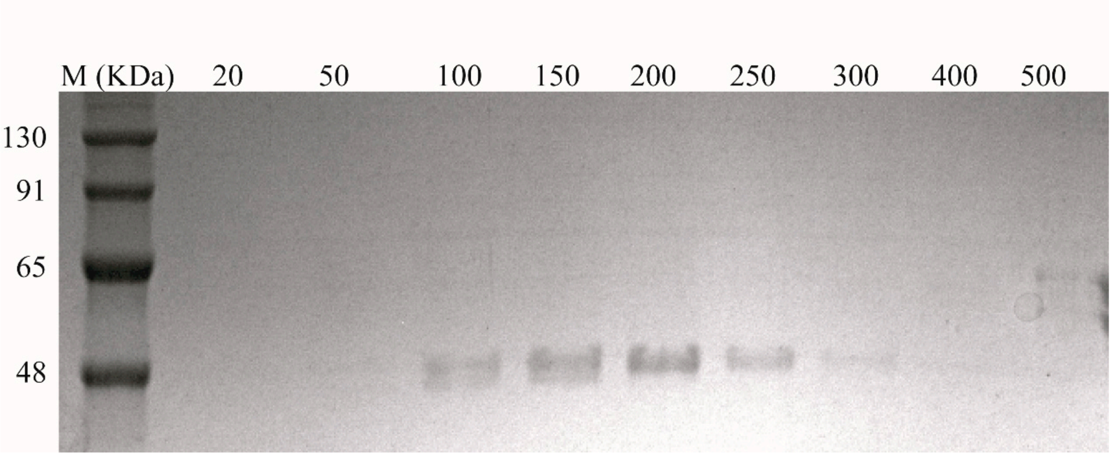

Figure S1. Gradient concentration (20,50,100,150,200,250,300,400,500 mM) illustrating imidazole purification.
